# The effect of sertraline with or without propranolol on panic attacks in women: a controlled clinical trial

**DOI:** 10.1186/s40780-025-00466-x

**Published:** 2025-07-04

**Authors:** Sahar Rouhzendeh, Zahra Mousavi, Sepideh Mashayekh-Amiri, Elnaz Shaseb, Seyedeh Tahereh Mirmolaei, Mojgan Mirghafourvand

**Affiliations:** 1 Student Research Committee, Department of Midwifery, Faculty of Nursing and Midwifery, Tabriz University of Medical Sciences, Tabriz, Iran; 2 Department of Psychiatry, Faculty of Medicine, Tabriz University of Medical Sciences, Tabriz, Iran; 3 Department of Midwifery, Faculty of Nursing and Midwifery, Golestan University of Medical Sciences, Gorgan, Iran; 4 Department of Clinical Pharmacy, School of Pharmacy, Tabriz University of Medical Sciences, Tabriz, Iran; 5 Reproductive Health Research Center, Department of Midwifery, School of Nursing and Midwifery, Tehran University of Medical Sciences, Tehran, Iran; 6 Social Determinants of Health Research Center, Tabriz University of Medical Sciences, Tabriz, Iran

**Keywords:** Panic attacks, Propranolol, Sertraline, Depression

## Abstract

**Background:**

The use of propranolol in the treatment of panic attacks has historically been widespread. However, there is insufficient precise evidence regarding the combination of propranolol with sertraline in this context. Panic attacks are a common health issue among women, significantly affecting their personal and social lives. Therefore, this study aimed to determine the effect of sertraline, with or without propranolol, on the severity of panic attack symptoms and depressive symptoms.

**Methods:**

This randomized controlled clinical trial included 60 women who attended a specialized university clinic in Tabriz. Participants were randomly assigned to either the sertraline with propranolol group (*n* = 30) or the sertraline with propranolol placebo group (*n* = 30) using block randomization method. Both groups received drugs for four weeks. Data were collected using the Panic Disorder Severity Scale– Self-Report (PDSS-SR) and the Beck Depression Inventory-Short Form (BDI-13). The Mann-Whitney U test was employed to compare outcomes between the two groups, and Wilcoxon tests were used for within-group comparisons.

**Results:**

Samples were enrolled from July 22, 2024, to February 11, 2025. The two groups exhibited no statistically significant differences in sociodemographic characteristics (*p* > 0.05). After the intervention, the mean score for the severity of panic attack symptoms in the sertraline with propranolol group was 6.6 (SD: 4.4), compared to 13.1 (SD: 5.4) in the sertraline with propranolol placebo group, indicating a statistically significant difference (*P* < 0.001). Additionally, the mean (SD) post-intervention depression score was 8.9 (4.8) in the sertraline with propranolol group and 15.5 (7.2) in the sertraline with propranolol placebo group, with the sertraline with propranolol group demonstrating a significantly lower mean depression score according to the Mann-Whitney U test (*p* = 0.001). Within-group comparisons also revealed a significant reduction in the severity scores for both panic attack symptoms and depression in the sertraline with propranolol group (*P* < 0.001).

**Conclusion:**

Based on the results of this trial, using propranolol alongside sertraline reduces the severity of panic attacks. Given these promising results, further studies in various settings are recommended to provide high-certainty evidence in this field.

**Trial registration:**

Iranian Registry of Clinical Trials (IRCT): IRCT20120718010324N70. Date of registration: 2022-08-31; URL: https://irct.behdasht.gov.ir/user/trial/65033/view.

## Background

The hormones associated with females significantly influence the lives of all women; an increase during reproductive years [1] or a decrease during menopause manifests various emotions and feelings [2]. Biological and neuroendocrine changes can sometimes negatively affect a woman’s perception of her physical and mental health [3]. Certain mental disorders, including panic disorders, are common among women [4], with a prevalence reaching 9.17%, and may be associated with stressful life events, depression, comorbid medical conditions, and functional impairment [5].

Panic disorder is a common mental health condition with a lifetime prevalence of 1.6–2.2% in the general population [6]. According to the Diagnostic and Statistical Manual of Mental Disorders (DSM-VI), panic disorder is characterized by recurrent and unexpected attacks, which are episodes of intense fear or severe discomfort that have a sudden onset and peak within 10 min, during which at least 4 out of 13 specified symptoms are experienced. These symptoms include increased heart rate, chest pain or discomfort, sweating, trembling, dizziness, heat sensations, fainting, shortness of breath, a feeling of choking, nausea, instability, lightheadedness, feelings of unreality or depersonalization (detachment from oneself), fear of losing control, fear of dying, and paresthesia (numbness or tingling) [7]. It has also been reported that panic disorder is associated with major depressive disorder, with a prevalence ranging from 24 to 88%, which increases the risk of suicide [8].

Available pharmacological treatments for panic disorder include tricyclic antidepressants, benzodiazepines, selective serotonin reuptake inhibitors (SSRIs), serotonin-norepinephrine reuptake inhibitors (SNRIs), and alpha- and beta-adrenergic agents such as propranolol and clonidine [9]. A 2021 systematic review comparing treatments for panic attacks in individuals with or without agoraphobia found that tricyclic antidepressants, benzodiazepines, monoamine oxidase inhibitors, selective serotonin reuptake inhibitors, and serotonin-norepinephrine reuptake inhibitors all led to improvements in panic attacks compared to placebo. Among these, tricyclic antidepressants and selective serotonin reuptake inhibitors were identified as the best treatments for panic disorder. Among the SSRIs, sertraline and citalopram were recognized as the most effective drugs due to their high recovery rates and fewer side effects [10]. Sertraline also has minimal effects on norepinephrine and dopamine reuptake, and research has shown it to have greater dopaminergic activity than other drugs in its class. Its mechanism of action makes it highly effective when used in the treatment of various psychiatric conditions, including depression and panic disorder [11]. Sertraline is classified as a selective serotonin reuptake inhibitor (SSRI) that selectively inhibits the reuptake of serotonin at the presynaptic membrane, thereby increasing the concentration of serotonin in the synaptic cleft and enhancing central 5-hydroxytryptaminergic neural activity. Serotonin plays a crucial role in regulating mood, personality, and wakefulness within the central nervous system, which is why blocking serotonin reuptake is highly beneficial in disorders such as depression and panic disorder [12].

Propranolol is another medication investigated for treating panic disorder. It is a non-selective beta-adrenergic receptor antagonist classified as an antiarrhythmic agent [13]. Although beta-blockers do not directly treat the psychological symptoms of anxiety, they help control physical symptoms such as palpitations, which may in turn form part of a positive feedback loop that reduces anxiety [14]. There has been a growing interest in prescribing propranolol for psychiatric issues. In a study by Turner and colleagues, its anxiolytic effects in reducing tachycardia caused by hyperthyroidism were noted [15]. The use of propranolol for the treatment of anxiety disorders is increasing, with it being approved in the UK [16], and prescribed off-label in other countries such as the United States [17] and Canada [18]. A 2016 systematic review aiming to assess the effect of propranolol on anxiety disorders found no statistically significant difference between benzodiazepines and propranolol in the short-term treatment of panic disorder [19]. On the other hand, results from a 2022 review study showed no evidence supporting the long-term effectiveness of propranolol in treating anxiety disorders other than panic disorder. Furthermore, this research was centered on a limited number of small studies that carry a substantial risk of bias regarding propranolol’s impact on anxiety disorders. Consequently, the quality of evidence for the effectiveness of propranolol is currently insufficient to support its routine use in treating fear and anxiety disorders [20]. Additionally, there are no clinical guidelines regarding when or how to use it [21].

Given the limited number of studies and insufficient evidence regarding the effectiveness of propranolol in treating panic disorder, coupled with the high prevalence of this condition among women and the established mechanisms of action of sertraline and propranolol, the present study aims to determine the effects of sertraline, with or without propranolol, in treating panic disorder in women.

## Methods

### Study design and participants

This was a double-blind, randomized clinical trial with two parallel arms. Participants, intervention administrators, care providers, and data collectors were all blinded to the type of intervention received by each participant.

The study population included women diagnosed with panic disorder by the investigator based on the Diagnostic and Statistical Manual of Mental Disorders (DSM-VI) criteria, who attended Sheikh al-Raees Clinic and the psychiatric outpatient clinic at Imam Reza Hospital in Tabriz. Exclusion criteria included substance or alcohol abuse; experiencing a stressful event (such as divorce, death of a first-degree family member, diagnosis of a terminal or hard-to-treat illness in a family member, or job loss) within the past three months; the presence of cardiac, pulmonary, diabetic, asthmatic, gastrointestinal, hepatic, hematologic, or endocrine disorders; and the use of antidepressants, antihistamines, barbiturates, narcotics, diazepam, amphetamines, or cocaine.

### Recruitment, randomization, and blinding

The women diagnosed with panic disorder, which is characterized by sudden and unexpected episodes of intense fear or discomfort that peak within 13 min and involve at least 3 of 13 specific symptoms—including increased heart rate, chest pain, sweating, trembling, dizziness, heat sensations, fainting, shortness of breath, choking sensation, nausea, instability, lightheadedness, feelings of unreality or depersonalization, fear of losing control, fear of death, and paresthesia were selected using a convenient sampling method. After careful screening with an inclusion and exclusion criteria checklist, eligible individuals who provided written informed consent were enrolled in the study. Following the baseline assessment, participants were randomly assigned to the sertraline plus propranolol group and the sertraline plus propranolol placebo group, according to the sequential allocation order. Allocation was done using a computer program (www.random.org) with a random block design of four and six blocks, at a 1:1 allocation ratio. To conceal the allocation, opaque and identical glass bottles were numbered consecutively. For each participant, two drug bottles were prepared: one containing sertraline and the other containing either propranolol or its placebo for a 4-week treatment period. Propranolol and its placebo had identical appearance characteristics (shape, color, smell, etc.). The principal investigator (PI) (SR, the first author) conducted participant recruitment and allocation into the groups. A person not involved in the sampling, intervention administration, care provision, or data collection conducted the sequence determination and preparation of the bottles.

### Intervention

The study participants underwent treatment that involved sertraline tablets in addition to propranolol or a placebo tablets. Propranolol was prescribed under the brand name Pranol^®^ (Propranolol hydrochloride tablet oral 10 mg). The administration of Propranolol or its placebo began at 10 mg during the first week and was increased to 20 mg starting from the second week. Sertraline was initiated at a dose of 25 mg in the first week, then raised to 50 mg in the second week, and further to 75 mg over the following two weeks. The duration of both medications was four weeks from the start of administration.

### Outcome and data collection

The primary outcomes were the frequency and severity of panic disorder, assessed using the Panic Disorder Severity Scale– Self Report (PDSS-SR). The secondary outcomes included depression scores and adverse events.

PDSS-SR is a new self-report diagnostic tool designed to assess panic disorder and monitor the severity of symptoms over the past week. Developed in 2002 by Patricia and colleagues, it consists of seven items that evaluate the frequency of panic attacks, distress during attacks, anticipatory anxiety, agoraphobic fear/avoidance, fear/avoidance of physical sensations related to panic, work impairment, and social dysfunction based on patient ratings. The scale uses a five-point range (0 = “not at all” to 4 = “most severe”), with higher scores indicating greater symptom severity. The total score ranges from 0 to 28 [22]. In the current study, Cronbach’s alpha was calculated to be 0.866.

The Beck Depression Inventory (BDI-13) Short Form was used to measure depression scores. This tool was designed by Beck in 1961, and its short form comprises 13 items that assess the global, behavioral, and cognitive symptoms of depression. Each item features four options, scored from 0 to 3, determining varying degrees of depression, from mild to severe. The maximum score for this test is 39, while the minimum is 0. A score of 0–4 indicates no depression, 5–7 indicates mild depression, 8–15 indicates moderate depression, and 16–39 indicates severe depression [23]. The Persian version of this tool has been psychometrically tested and validated in Iran [24].

A side effect checklist was used to record adverse events during the study. The assessment and follow-up of participants were conducted in two phases: the first assessment was before entry into the study, and the second assessment was 4 weeks after entry into the study. To ensure medication adherence and emphasize its importance, participants were contacted weekly via telephone to remind them to take their medications.

The baseline and sociodemographic questionnaire, Panic Disorder Severity (Self-Report Form), and Beck Depression Inventory were completed through interviews with the participants.

### Sample size

The sample size was calculated based on the variable of panic attack severity using G-Power software. According to the results of Forsell et al.s’ study [25] regarding the panic attack severity variable, with M_1_ = 11.2, M_2_ = 7.28, SD_1_ = 4.9, SD_2_ = 4.7, a two-sided α = 0.05, and 80% power, the required sample size was determined to be 25 participants. Considering a 20% attrition rate, the final sample size was 30 participants per group.

### Data analysis

The data were analyzed using SPSS version 26. Independent t, chi-square, chi-square for trend, and Fisher’s exact tests were used to compare the groups regarding socio-demographic characteristics. The normality of the distribution of quantitative data (panic and depression scores) was assessed using the Kolmogorov-Smirnov test. Given the non-normal distribution of quantitative data, the Mann-Whitney U test was used to compare the groups regarding the median of quantitative outcomes. An intragroup comparison was performed using the Wilcoxon Signed Ranks Test. All analyses were conducted based on an intention-to-treat approach. A *p*-value of < 0.05 was considered statistically significant.

## Results

**Fig. 1:**
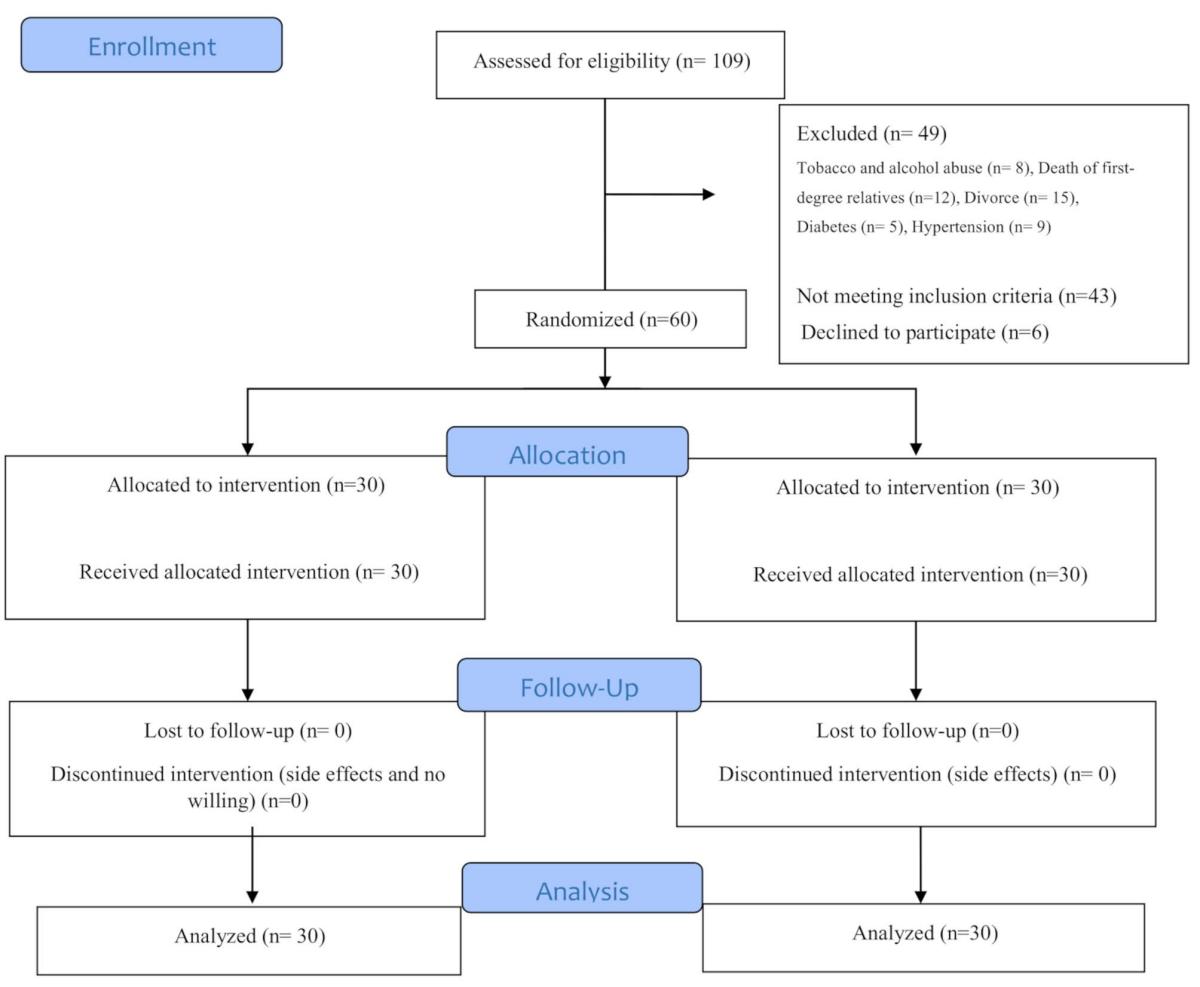
Flow chart of the study

Participants were selected from July 22, 2024, to February 11, 2025. Of the 109 women assessed, 66 met the inclusion criteria; however, six declined participation. Eight women were excluded due to substance abuse (alcohol and tobacco), 12 due to the death of immediate family members, 15 due to divorce, five due to diabetes, and nine due to high blood pressure. All 30 women allocated to each group (one month after the intervention) were followed up (Fig. 1).

The two groups showed no significant differences in sociodemographic and obstetric characteristics. The mean age of women in the sertraline with propranolol group was 40.6 years (SD = 9.9), while in the sertraline without propranolol group, it was 38.2 years (SD = 8.7) (Table 1).

**Table 1:** Baseline characteristics of participants by study group

Variable	Sertraline with propranolol (*n* = 30)		Sertraline with propranolol placebo (*n* = 30)	*P*-value
	Mean (SD)		Mean (SD)	
**Age (years)**	40.6 (9.9)		38.2 (8.7)	0.323^a^
	**Number (percent)**		**Number (percent)**	
**Education**				0.054^c^
Illiterate	2 (6.7)		0 (0.0)	
Primary school	9 (30.0)		6 (20.0)	
Secondary school	6 (20.0)		4 (13.3)	
High school	5 (16.7)		4 (13.3)	
Diploma	5 (16.7)		12 (40.0)	
University	3 (10.0)		4 (13.3)	
**Job cat**				0.104^d^
Housewife	27 (90.0)		21 (70.0)	
Employed	3 (10.0)		9 (30.0)	
**Sufficiency of family income for expenses**				0.610^c^
Insufficient	3 (10.0)		3 (10.0)	
Relatively sufficient	23 (76.7)		22 (73.3)	
Completely sufficient	4 (13.3)		5 (16.7)	
**Husbands job**				0.235^b^
Unemployed	2 (6.7)		0 (0.0)	
Worker	4 (13.3)		1 (3.3)	
Employee	3 (10)		5 (16.7)	
Freelance job	21 (70)		24 (80)	
**Life satisfaction**				
High		2 (6.7)	0 (0.0)	0.351^c^
Moderate		17 (56.7)	16 (53.3)	
Low		11 (36.7)	14 (46.7)	
**Number of pregnancies**				0.206^d^
1	10 (33.3)		9 (30.0)	
2	10 (33.3)		16 (53.3)	
≥3	10 (33.3)		5 (16.7)	
**Number of births**				0.501^d^
1	13 (43.3)		15 (50.0)	
2	9 (30.0)		12 (40.0)	
≥3	8 (26.7)		3 (10.0)	
**Past medical history**				0.612^b^
None	27 (90.0)		27 (90.0)	
Hypertension	3 (10.0)		1 (3.3)	
Other	0 (0.0)		2 (6.6)	

Abbreviations: SD, standard deviation. ^a^Independent t test; ^b^Fisher’s exact test,^c^Linear-by-linear association, ^d^Pearson chi-square test

### Primary outcome

The mean panic attack severity score before the intervention in the sertraline with propranolol group was 18.9 (SD = 3.6); in the sertraline with propranolol placebo group, it was 18.7 (SD = 4.0). Based on the Mann-Whitney U test, no statistically significant difference existed between the groups in the mean panic attack severity score before the intervention (*P* = 0.766). One month after the intervention, the mean (SD) panic attack severity score in the sertraline with propranolol group was 6.6 (4.4), while in the sertraline without propranolol group, it was 13.1 (5.4). According to the Mann-Whitney U test, the panic attack severity score one month after the intervention was significantly lower in the sertraline with propranolol group compared to the sertraline with propranolol placebo group (*P* < 0.001). Intragroup comparison results indicated a significant reduction in the panic attack severity score within the sertraline with propranolol group (*P* < 0.001) (Table 2).

**Table 2:** Comparison of panic attacks and depression scores between study groups

Variable	Sertraline with propranolol (*n* = 30)		Sertraline with propranolol placebo (*n* = 30)		Intergroup comparison (*P* value)^a^
	Mean (SD)	Median (per 25 to 75)	Mean (SD)	Median (per 25 to 75)	
**Severity of panic attacks (Score range: 0 to 28)**					
Before intervention	18.9 (3.6)	18.5 (17.0 to 22.0)	18.7 (4.0)	20.0 (18.0 to 20.2)	0.766
One month after intervention	6.6 (4.4)	5.5 (3.0 to 10.0)	13.1 (5.4)	15.0 (10.2 to 17.0)	˂0.001
**Intragroup comparison** (*P*-value)^b^	< 0.001		< 0.001		
**Depression (Score range: 0 to 39)**					
Before intervention	24.8 (7.9)	26.5 (19.0 to 31.0)	22.4 (7.3)	24.0 (19.7 to 27.0)	0.046
One month after intervention	8.9 (4.8)	8.0 (6.0 to 11.0)	15.5 (7.2)	18.0 (8.0 to 22.2)	0.001
**Intragroup comparison** (*P*-value) ^b^	< 0.001		< 0.001		

^a^ Mann‒Whitney U test; ^b^Wilcoxon Signed Ranks Test

### Secondary outcomes

Before the intervention, the mean (SD) depression score in the sertraline with propranolol group was 24.8 (7.9), while in the sertraline with propranolol placebo group, it was 22.4 (7.3). According to the Mann-Whitney U test, the mean depression score before the intervention was significantly higher in the sertraline with propranolol placebo group compared to the sertraline with propranolol group (*P* = 0.046). One month after the intervention, the mean (SD) depression score in the sertraline with propranolol group decreased to 8.9 (4.8), whereas in the sertraline with propranolol placebo group, it was 15.5 (7.2). Based on the Mann-Whitney U test, the mean depression score one month after the intervention was significantly lower in the sertraline with propranolol group compared to the sertraline with propranolol placebo group (*P* = 0.001). Intragroup comparison results revealed a significant reduction in the depression score within the sertraline with propranolol group (*P* < 0.001) (Table 2).

### Adverse effects

The participants reported no adverse effects during the study.

## Discussion

The results of this trial demonstrated that the simultaneous use of sertraline and propranolol leads to a reduction in panic attacks and symptoms of depression in women. The basline depression score in the intervention group was higher than the control group, which was a very small difference (2.5 points). However, the results of the intragroup comparison showed a significant reduction in depression score in the sertraline with propranolol group. Also, in this study, alongside statistically analyzing the observed changes in PDSS-SR and BDI scores, we also assessed the effect size using Cohen’s *d*, which yielded values of *d* = − 1.31 for panic (PDSS-SR) and *d* = − 1.08 for depression (BDI). These results indicate clinically very large effect sizes.

To our knowledge, there is currently insufficient and precise evidence regarding the impact of beta-blockers, including propranolol, on panic attacks and anxiety disorders. Furthermore, no study has been found that investigated the use of sertraline in combination with propranolol for treating panic attacks, making this study the first of its kind in this regard.

In the crossover study by Kathol et al. (1980), it was shown that the use of propranolol, compared to placebo, did not result in a significant reduction in the severity of panic attacks [26]. In the clinical trial by Munjack et al. (1989), comparing the use of alprazolam with propranolol and placebo, no significant differences were found in the severity and frequency of panic attacks among the groups studied [27]. Similarly, the crossover study by Noyes et al. (1980) showed that the use of diazepam, compared to propranolol, did not significantly reduce the number of panic attacks [28]. In each of these studies, the effect of propranolol alone on panic attacks was assessed, without combining it with another medication. According to our searches, we did not find any recent clinical trials on the effect of propranolol on panic attacks, and the available evidence is somewhat outdated [27–30]. However, in the current study, propranolol was administered alongside sertraline.

There is evidence suggesting that beta-blockers are increasingly prescribed alongside antidepressant medications as a combination treatment, as they are believed to enhance the effects of antidepressants [31]. However, conflicting results exist regarding this approach. In a clinical trial by Hirschmann et al. in 2000, it was shown that the use of a beta-blocker alongside the antidepressant fluoxetine significantly reduced panic attacks [32]. In another trial conducted by Stein et al. in 2000, the beta-blocker did not demonstrate superiority over placebo in enhancing the effects of paroxetine on social anxiety symptoms [33].

A systematic review aimed at evaluating the effectiveness of oral propranolol versus placebo as a treatment option for reducing anxiety included four studies related to panic disorder with or without agoraphobia (130 participants), two studies on specific phobias (37 participants), one study on social phobia (16 participants), and one study on post-traumatic stress disorder (PTSD) (19 participants). Of the four trials on panic disorder, three were eligible for meta-analysis. These meta-analyses did not find a statistically significant difference between the effects of propranolol and benzodiazepines for the short-term treatment of panic disorder with or without agoraphobia. Additionally, no evidence was found for the effects of propranolol on the severity of PTSD symptoms through the inhibition of memory reconsolidation. As a result, the quality of evidence for the effectiveness of propranolol currently does not support its routine use in the treatment of any anxiety disorders [19]. On the other hand, there has been a more than 50% increase in the use of beta-blockers for anxiety disorders among adults [16], with qualitative evidence suggesting that beta-blockers are preferred by patients who do not want to take typical psychotropic medications or are concerned about the long-term effects of antidepressants [34].

A recently published systematic review by Archer et al. (2024) found no evidence supporting the beneficial effects of beta-blockers compared to placebo or benzodiazepines in patients with social phobia or panic disorder, with or without agoraphobia, regarding anxiety symptoms or the number of panic attacks. This review identified only a small number of eligible studies (*n* = 10), with just five included in the meta-analysis. Consequently, the effect estimates and confidence intervals may not be reliably interpreted. Furthermore, many previous studies had small sample sizes, which likely reduced the power to detect meaningful differences between groups. Additionally, several of the included studies were conducted over thirty years ago, which may limit the applicability of the findings to the present day, as clinical practices have evolved. The quality of the included studies was poor, with nearly all of them carrying a high (or unclear) risk of bias in several areas [21].

Propranolol was first discovered in the early 1920s, and its anxiolytic effects were noticed somewhat incidentally [35]. These effects may be linked to its ability to induce amnesia for emotional memory processes [36]. Additionally, a clinical trial demonstrated propranolol selectively interferes with long-term emotional memory performance by blocking the β-adrenergic system while leaving non-emotional memories unaffected [37]. Researchers attribute these effects of propranolol to its ability to regulate norepinephrine levels in the brain. As a drug that can cross the blood-brain barrier, it directly impacts the central nervous system [38–40]. Therefore, propranolol may have the capacity to reduce the recall of emotional and neutral stimuli, diminishing their emotional impact [41].

This study boasts several strengths, including a low likelihood of clinical trial biases. These include selection bias minimized by a suitable randomization process, performance and diagnostic bias mitigated through a double-blind design, and reporting bias addressed by documenting and analyzing all pre-planned primary and secondary outcomes as intended. Additionally, the absence of sample attrition concerning the primary and secondary outcomes reduces the likelihood of attrition bias for these results. All sampling stages were conducted by a psychiatry specialist and the principal investigator (SR), including the assessment of eligibility criteria and intervention follow-up.

However, the study’s limitations include the small sample size and the restriction to a female population. Therefore, it is recommended that future trials be conducted in various settings and with different dosages of the medications to enhance the generalizability of the results. It is also recommended that future studies be designed with a similar method to compare anxiety symptoms and long-term effects of this type of intervention.

## Conclusion

Based on the results of this trial, the combination of propranolol and sertraline leads to a reduction in the severity of panic attacks and depressive symptoms. Given these promising results, further studies in different settings are recommended to provide high-certainty evidence in this area.

## Data Availability

The datasets generated and/or analyzed during the current study are not publicly available due to limitations of ethical approval involving the patient data and anonymity but are available from the corresponding author at reasonable request.

## Abbreviations

PDSS-SR: Panic Disorder Severity Scale– Self-Report
BDI-13: Beck Depression Inventory
IRCT: Iranian Registry of Clinical Trials
DSM-VI: Diagnostic and Statistical Manual of Mental Disorders
SSRIs: Selective serotonin reuptake inhibitors
SNRIs: Serotonin-norepinephrine reuptake inhibitors
PI: Principal investigator
SD: Standard Deviation
PTSD: Post-traumatic stress disorder
